# Case of Small Pieces of Bone Discharged from Ulcers in the Sternum of a Child

**Published:** 1815-01

**Authors:** John Mitchell

**Affiliations:** Mile's End, near Stockport


*For the Medical and Physical Journal.*

### *Case of Small Pieces of Bone discharged from Ulcers in the Sternum of a Child;* by J. Mitchell, M.D.

SOME time in July, a weaver brought his child, a female, between six and seven years of age, for my advice and inspection. In examining the child's case, I observed a large cicatrix on the skin over the sternum, and at the end of one of the ribs that joined to it, on the right side, a round white-edged ulcer, the orifice about the size of a small hazel-nut; below the breast, on the same side, there was another ulcerous orifice, of the same size and appearance, at the fore part of one of the true ribs.

Supposing the child's complaint to be scrophulous, I inquired what effect the ulcers had upon the girl's health: he made answer that she appeared hearty; she followed her work of dyeing cotton for the spinners; when from her work, she ran about and played with other children, and had a good appetite for her victuals; but that pieces of bones were protruding from the ulcers, two or three in the fortnight. He opened a paper, and showed me more than a dozen of pieces, all of ribs, some of which were above three inches long, having curvatures, but without any periostium. He showed me also a square bone, which he said came from the mark on her breast: it was the entire middle portion of the sternum, near two inches long, and a quarter of an inch thick. All the pieces had an old look, as if they had been gathered from some burying ground. I asked if there were any discharge of matter from the ulcers? He replied, there was little or none, unless when the pieces were shooting out, which which he said was surprising to behold, for they came out by degrees without any pain, as if coming from a sheath. The ulcers were kept without any dressing. I pressed with my finger upon the cicatrix over the sternum, but there was no deficiency of bone to my feeling; along the course of the ribs, where the ulcers were, all seemed firm to the pressure. I told her father, that, according to my judgment, nothing could be done for his child; that he must wait till nature and her growth might make a change in her favour. About six weeks since, he overtook me in the street, and said his child had many pieces of bone discharged from her breast since he last had seen me, and that she continued in other respects in good health.

If the above case be worthy a place in your valuable Journal, it is at your service. I would not have troubled you had it not appeared to me to be a new disease of the bones. Perhaps other practitioners may have seen similar cases: if they have, it is more than I have ever heard. Most likely it originates from scrophulous affection of the periostium. JOHN MITCHELL, M.D.

*Mile's End, near Stockport;*

*Oct.* 10, 1814.

